# Clinical implications of pathological features of primary membranous nephropathy

**DOI:** 10.1186/s12882-018-1011-5

**Published:** 2018-08-28

**Authors:** Xiao-dan Zhang, Zhao Cui, Mu-fan Zhang, Jia Wang, Yi-miao Zhang, Zhen Qu, Xin Wang, Jing Huang, Fang Wang, Li-qiang Meng, Xu-yang Cheng, Su-xia Wang, Gang Liu, Ming-hui Zhao

**Affiliations:** 1 Renal Division, Key Laboratory of Renal Disease, Ministry of Health of China, Key Laboratory of CKD Prevention and Treatment, Ministry of Education of China, Peking University First Hospital, Institute of Nephrology, Peking University, Beijing, 100034 People’s Republic of China; 2 Laboratory of Electron Microscopy, Pathological Centre, Peking University First Hospital, Beijing, People’s Republic of China; 3 Peking-Tsinghua Center for Life Sciences, Beijing, 100871 PR China

**Keywords:** Membranous nephropathy, Pathology, Tubulointerstitial injury, Prognosis

## Abstract

**Background:**

The clinical outcome varies considerably in primary membranous nephropathy (pMN). Risk factors for kidney prognosis include ageing, male gender, persistent heavy proteinuria, decreased eGFR at presentation, persistent elevation of anti-PLA2R antibodies, no remission, and so on. It was controversial whether the histopathological features of pMN could predict treatment response and kidney outcome.

**Methods:**

A retrospective study was conducted in 371 patients with biopsy-proven pMN. Pathological parameters included immunofluorescence staining, membranous Churg’s stages, sclerosis, crescent, focal segmental sclerosis lesion, chronic and acute tubulointerstitial injury. The fluorescence intensity was determined: 0, negative; 1, weak; 2, moderate; 3, strong; 4, glaring. Chronic tubulointerstitial injury was graded by the involved area: 0, 0–5%; 1, 6–25%; 2, 26–50%; 3, > 50%.

**Results:**

We found that patients with higher intensity of C3 staining, advanced membranous stage, and more severe chronic tubulointerstitial injury presented with higher positivity rate of anti-PLA2R antibodies, higher levels of urinary protein excretion and serum creatinine, and lower level of serum albumin. Univariate Cox regression analysis showed that severe (grade = 3) chronic tubulointerstitial injury was a risk factor to the kidney outcome of ESKD (HR = 61.02, 95%CI, 7.75–480.57, *P* < 0.001) and over 50% reduction of eGFR (HR = 4.43, 95%CI, 1.26–15.6, *P* = 0.021). Multivariate analysis demonstrated it as an independent risk factor to ESKD (HR = 25.77, 95% CI, 1.27–523.91, *P* = 0.035). None of the pathological parameters exerted any influence on treatment response (*P* > 0.05).

**Conclusions:**

We found the prognostic role of chronic tubulointerstitial injury to the kidney outcome of pMN. This study highlighted the value of kidney biopsy under the widespread usage of anti-PLA2R antibodies for diagnosis and prognosis.

**Electronic supplementary material:**

The online version of this article (10.1186/s12882-018-1011-5) contains supplementary material, which is available to authorized users.

## Background

Primary membranous nephropathy (pMN) is the most common cause of adult-onset nephrotic syndrome [1]. The diagnosis of pMN depends on the pathological manifestation which is characterized by the presence of subepithelial immune complexes, diffuse thickening of glomerular basement membrane by light microscopy, and granular deposit of IgG and complement along the periphery of glomerular capillary loops by immunofluorescence. M-type phospholipase A2 receptor (PLA2R), a transmembrane protein expressed on podocytes, has been defined as the major autoantigen of this disease [2]. The specific antigen is co-localized with IgG4 in the subepithelial immune deposits. Circulating antibodies against PLA2R were detected in about 70% of patients. It has been documented that the presence of anti-PLA2R has close associations with clinical parameters, thus the antibody has been utilized as a good biomarker in clinical practice [2].

The clinical consequence of pMN varies considerably. If untreated, about one-third of patients undergo spontaneous remission [3], especially those with absent or low levels of anti-PLA2R antibodies [4], another one-third of patients progress to end-stage kidney disease (ESKD) over 10 years [5], and the remainder develop non-progressive chronic kidney disease (CKD). However, if with proper management, only 10% or less will develop ESKD over the subsequent 10 years [6].

Various risk factors affect kidney prognosis. The established ones include age, male gender, persistent heavy proteinuria, decreased GFR on presentation, increased excretion of β2 microglobulin and increased urinary excretion of C3dg and C5b-9 [3, 7–12]. Persistent elevation of anti-PLA2R levels after therapy is a significant indicator to poor prognosis [9, 10]. However, the findings on histologic variables are controversial. The advanced stage of glomerular lesions detected by electron microscopy has been suggested to correlate with worse renal survival in some but not all studies [13]. Several researches have reported that chronic interstitial fibrosis and tubular atrophy are independent predictors of progressive renal failure [14]. In addition, focal segmental glomerular sclerosis superimposed on MN has been found as indicator of a poor prognosis [15], while its importance was not substantiated in a retrospective analysis of a large cohort from the University of Toronto [16].

Using a large cohort of patients with biopsy-proven pMN, we have demonstrated that the presence of crescents showed undesirable response to the treatments and worse kidney outcomes [17]. In the current study, we investigated the histopathologic features of pMN patients with credible medical records of therapeutic responses and kidney outcomes during follow-up, aiming to clarify the clinical indications of pathological parameters of pMN.

## Methods

### Study population

A total of 371 consecutive patients with kidney biopsy-proven pMN, who were diagnosed and followed up in Peking University First Hospital from 2008 to 2016, were reviewed retrospectively in this study. Patients with known secondary MN, such as hepatitis B/C virus infection, lupus, malignancy, rheumatoid arthritis, medications, and heavy metal poisoning, were excluded. Clinical data were collected from medical records at the time of diagnosis as well as during follow-up.

The research was in compliance of the Declaration of Helsinki and was approved by the ethics committee of Peking University First Hospital. Written informed consent was obtained for sampling tissue and blood.

Estimated glomerular filtration rate (eGFR) was calculated from serum creatinine levels using the Modification of Diet in Renal Disease Study equation adjusted for Chinese populations: eGFR = 175× (serum creatinine)^1.234^ × age^0.179^ × 0.79 (if female) [18].

### Kidney biopsies

Kidney biopsy was performed at the time of diagnosis in all patients. Renal specimens were evaluated with direct immunofluorescence, light microscopy, and electron microscopy, as described previously [19]. The methods were performed according to the standard operating procedure at our center [19]. Direct immunofluorescence for IgG subclasses was performed after 2014, using mouse anti-IgG1, -IgG2, -IgG3 and -IgG4 monoclonal antibodies (clone no. 4E3, HP6014, HP6050, HP6025; Southern Biotech, Birmingham, AL) at 1:100, as reported previously [20].

The fluorescence intensity of IgG, IgM, IgA, C3, C1q, and IgG subclass was determined using a semi-quantitative scale of 0 to 4: 0, negative; 1, weak staining; 2, moderate staining; 3, strong staining; 4, glaring staining.

Glomerular MN lesions were classified into four stages according to the Ehrenreich and Churg’s classification criteria. The chronic tubulointerstitial injury was defined as tubular atrophy and interstitial fibrosis, while the acute tubulointerstitial injury included the loss of tubular brush border and interstitial mononuclear cell infiltration. The scoring was graded semi-quantitatively from 0 to 4: 0, 0–5%; 1, 6–25% of interstitium involved; 2, 26–50% of interstitium involved; 3, > 50% of interstitium involved.

### Detection of circulating anti-PLA2R antibodies and anti-THSD7A antibodies

Circulating anti-PLA2R antibodies and circulating anti-thrombospondin type 1 domain containing 7A (THSD7A) antibodies were detected using commercial ELISA kits (EUROIMMUN AG, Lübeck, Germany) and immunofluorescence assay kit (EUROIMMUN AG, Lübeck, Germany) separately, following the standard instructions [17, 21].

### Treatment and follow-up

The use of corticosteroids and immunosuppressive agents, and the definitions of remission and relapse were in compliance with the 2012 KDIGO (Kidney Disease: Improving Global Outcomes) guideline for glomerulonephritis [22].

For evaluation of the renal outcomes, the primary endpoint was ESKD; the second endpoint was renal dysfunction, defined as eGFR reduction by more than 50% from the baseline (at the time of kidney biopsy) and the final eGFR being less than 60 ml/min/1.73m^2^.

### Toronto risk score

Toronto Risk Score by Cattran, et al. [23] was applied in this study. Toronto Risk Score = e^x^/(1 + e^x^), x = 1.26 + 0.3*persistent proteinuria-0.3*slope creatinine clearance-0.05*initial creatinine clearance; creatinine clearance was presented as eGFR; initial creatinine clearance was collected at baseline; the slope of creatinine clearance was calculated during the first 6 months. Persistent proteinuria was the minimum value of 24-h urinary protein excretion in the first six-month. [23, 24]

### Statistical analysis

Statistical analysis was performed using the SPSS statistical software package, version 13.0 (SPSS Inc., Chicago, IL). Normally distributed variates were expressed as the mean ± SD, and continuous variates of unnormal distribution were expressed as the median with interquartile range (IQR). Categorical variables were expressed as absolute values and percentages. For continuous variates, comparisons between two groups were performed using Student’s t test for normally distributed data and Wilcoxon rank-sum test for nonparametric data; comparisons among three or more groups were performed using one-way analysis of variance (ANOVA) for normally distributed data and Kruskal-Wallis test for nonparametric data. For categorical variables, Chi-square, Fisher exact test or Pearson Chi-square test were performed appropriately. If the differences among multi-groups were significant, SNK or Dunnett’s test was used to analyze differences between each pair of groups. Risk factors for no-remission after treatments were analyzed using Logistic regression model. Kaplan-Meier curves were used to analyze the kidney outcomes of patients. The predictors for kidney dysfunction were assessed using Cox regression model. Statistically significant parameters (*P* < 0.10) were enrolled into the Multivariate Cox regression models. Results were expressed as odds ratio (OR) or hazard ratio (HR) with 95% confidence interval (CI). All statistical analyses data were 2-tailed and the level of significance was set at 0.05.

## Results

### Clinical and pathological data

Three hundred seventy-one consecutive patients with kidney biopsy-proven pMN were reviewed in this study. The clinical and pathological data are shown in Table 1.

**Table 1:** Clinical characters of the patients with pMN

	*N* = 371
Age (years)	53, 43–61
Gender (M/F)	207/164
Nephrotic syndrome, n (%)	255 (68.7%)
Proteinuria (g/24 h)	4.0, 2.3–6.4
Serum albumin (g/L)	27.5 ± 6.1
Hematuria, n (%)	208 (56.1%)
Hypertension, n (%)	187 (50.4%)
Serum creatinine (μmol/L)	65.4, 54.0–80.0
eGFR (ml/min per 1.73m^2^)	114.2, 92.6–143.2
Anti-PLA2R antibody positivity, n (%)	243 (65.5%)
Anti-PLA2R antibody level ^a^(U/mL)	77.8, 34.9–200.0
Anti-THSD7A antibody positivity, n (%)	4/295 (1.4%)
Pathological features	
Immunofluorescence	
IgG deposit, n (%)	367 (98.9%)
IgG1, n (%)	151/171 (88.3%)
IgG2, n (%)	150/171 (87.7%)
IgG3, n (%)	126/171 (73.7%)
IgG4, n (%)	167/171 (97.7%)
IgA deposit, n (%)	103 (27.8%)
IgM deposit, n (%)	161 (43.4%)
C3 deposit, n (%)	351 (94.6%)
C1q deposit, n (%)	151 (40.7%)
Glomerular lesion	
Stage I, n (%)	193 (52.0%)
Stage II, n (%)	159 (42.9%)
Stage III, n (%)	19 (5.1%)
Stage IV, n (%)	0 (0%)
Global sclerosis (%)	5.1, 3.0–7.4
Crescent (%)	4.2, 2.7–5.1
Focal segmental glomerular sclerosis (%)	5.3, 4.2–6.3
Chronic tubulointerstitial injury	
Grade = 0	100 (27.0%)
Grade = 1	56 (15.1%)
Grade = 2	207 (55.8%)
Grade = 3	8 (2.1%)
Acute tubulointerstitial injury	32 (8.6%)
Treatments	
ACEI/ARBs only, n (%)	117 (31.5%)
Immunosuppressive therapies, n (%)	254 (68.5%)
Cyclophosphamide + corticosteroids	138/254 (54.3%)
Calcineurin inhibitor w/o corticosteroids	116/254 (45.7%)
Rituximab	0 (0%)
Treatment responses	
Complete remission, n (%)	168 (45.3%)
Partial remission, n (%)	157 (42.3%)
No remission, n (%)	46 (12.4%)
Relapse, n (%)	90/325 (27.7%)
Follow-up duration (months)	26.9, 13.9–45.4
ESKD, n (%)	8 (2.2%)
eGFR reduction > 50% from baseline, n (%)	67 (18.1%)

^a^Antibody level was calculated only for those over the cut-off value of 20 U/mL. Continuous and normally distributed variables were presented as mean ± SD; continuous and non-normally distributed variables were presented as median, IQR; categorical variables were presented as number (%)

Three hundred sixty seven (98.9%) patients presented with granular staining of IgG and 351 (94.6%) patients showed C3 staining along the glomerular capillary walls (Table 1). They all had immune deposit on the epithelial area by electron microscopy. IgG subclasses staining was available in 171 consecutive patients after 2014. Among them, IgG4 deposit was shown in 167 (97.7%) patients and IgG4 dominance was in 154 (90.1%) patients.

**Fig. 1:**
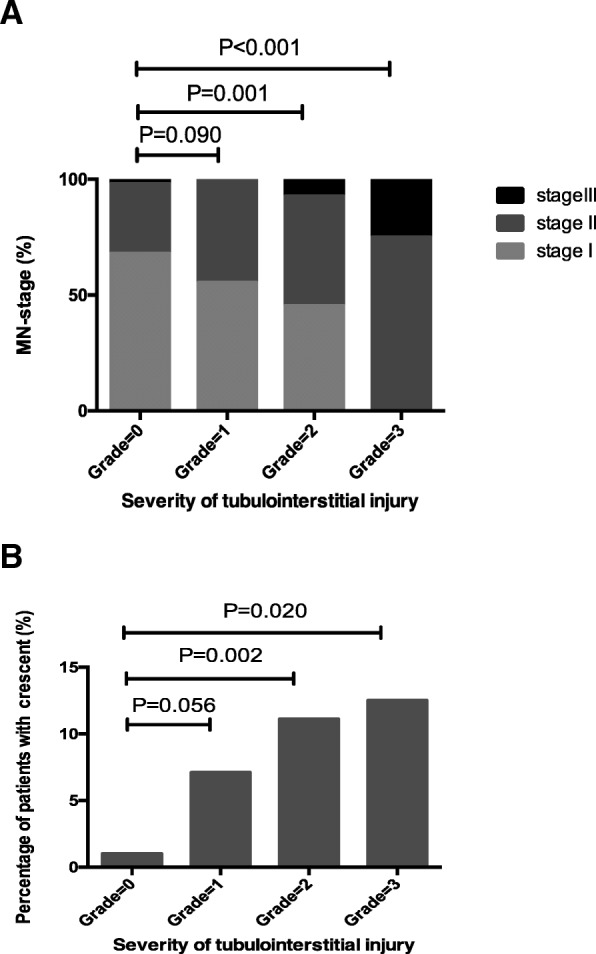
The comparison of pathological parameters in the pMN patients with different severity of tubulointerstitial injury. With the deterioration of tubulointerstitial injury, the stage of membranous lesion became more advanced (**a**) and the prevalence of crescent formation was increasing (**b**)

The stage of membranous lesion was parallel with the intensity of C3 deposition (*r* = 0.147, *P* = 0.005) and the grades of chronic tubulointerstitial injury (*r* = 0.247, *P* < 0.001). There were 48 (12.9%) patients having global sclerosis with a median percentage of 5.1 (3.0–7.4) %, and 19 (5.1%) patients having focal segmental sclerosis with a median percentage of 5.3 (4.2–6.3) %. Crescents were shown in 29 (7.8%) patients with a median percentage of 4.2 (2.7–5.1) %. The global sclerosis (7.0% vs. 7.1% vs. 17.4% vs. 12.5%, *P* = 0.030) and crescent (1.0% vs. 7.1% vs. 11.1% vs. 12.5%, *P* = 0.005) were more frequently shown in the patients with severer chronic tubulointerstitial injury (Fig. 1).

### The associations between pathological and clinical features

According to the increase of C3 staining intensity (Table 2), the patients presented with higher positivity rate of serum anti-PLA2R antibodies, higher level of urinary protein excretion, lower level of serum albumin, and higher level of serum creatinine. There was no difference on clinical features among the patients with different intensity of IgG staining (*P* > 0.05). The patients with IgG4 subclass dominant presented with higher level of eGFR [115.3 (97.8–146.2) vs. 92.0 (73.1–126.4) ml/min/1.73m^2^, *P* < 0.001].

**Table 2:** Comparisons of clinical characteristics of pMN patients with different intensity of C3 deposit

	C3 – (*n* = 20)	C3 1+ (*n* = 114)	C3 2+ (*n* = 186)	C3 3+ (*n* = 51)	*P*
Age (year)	53 (48–60)	53 (40–60)	53 (43–62)	52 (47–61)	0.822
Gender (M/F)	11/9	61/53	98/88	37/14	0.079
Nephrotic syndrome, n (%)	11 (55.0%)	67 (58.8%)	133 (71.5%)	44 (86.3%)	**0.002**
Proteinuria (g/24 h)	3.6 (2.3–7.1)	3.4 (2.3–5.8)	4.0 (2.2–6.0)	6.2 (3.5–9.6)	**0.001**
Serum albumin (g/L)	30.1 ± 8.7	29.7 ± 5.9	26.7 ± 5.5	24.4 ± 5.7	**< 0.001**
Microscopic hematuria, n (%)	4 (20.0%)	59 (52.7%)	112 (60.5%)	33 (66.0%)	**0.002**
Serum creatinine (μmol/L)	62.5 (50.0–80.8)	62 (53.7–79.3)	66.0 (53.0–79.5)	71.0 (62.6–93.4)	**0.048**
eGFR (ml/min per 1.73m^2^)	120.0 (99.2–143.4)	116.9(99.9–151.2)	115.2 (90.5–142.5)	102.6(80.4–127.0)	0.071
Anti-PLA2R antibody positivity, n (%)	11 (55.0%)	58 (51.3%)	136 (73.5%)	38 (74.5%)	**< 0.001**
Anti-PLA2R antibody level (U/mL)	76.3 (58.2–160.7)	97.5 (52.6–186.2)	117.8 (50.8–235.7)	135.3 (45.0–277.1)	0.653
Anti-THSD7A antibody positivity, n(%)	0 (0.0%)	2 (2.2%)	2 (1.3%)	0 (0.0%)	0.741
Hypertension, n (%)	8 (40.0%)	62 (54.4%)	88 (47.3%)	29 (56.9%)	0.369
Hemoglobin (g/L)	131.0 (119.0–144.0)	137.5 (128.0–151.0)	137.0 (126.0–149.0)	133.0 (115.3–143.5)	0.059

Continuous and normally distributed variables were presented as mean ± SD; continuous and non-normally distributed variables were presented as median, IQR; categorical variables were presented as number (%)

The numbers in boldface are statistically significant

With the aggravation of MN stages from I to III (Additional file 1: Table S1), the patients presented with higher positivity rate of serum anti-PLA2R antibodies, higher level of urinary protein excretion, and lower level of eGFR.

With the deterioration of tubulointerstitial injury from grade 0 to 3 (Table 3), the patients became older, and presented with higher positivity rate of serum anti-PLA2R antibodies, higher level of urinary protein excretion, lower level of serum albumin, lower level of eGFR, higher percentage of hypertension, and lower level of hemoglobin.

**Table 3:** Comparisons of clinical characteristics of pMN patients with different severity of chronic tubulointerstitial injury

	Grade = 0 (*n* = 100)	Grade = 1 (*n* = 56)	Grade = 2 (*n* = 207)	Grade = 3 (*n* = 8)	*P*
Age (year)	47 (32–58)	54 (44–62)	56 (47–63)	58 (50–70)	**< 0.001**
Gender (M/F)	49/51	34/22	121/86	3/5	0.252
Nephrotic syndrome, n (%)	51 (51.0%)	37 (66.1%)	161 (77.8%)	6 (75.0%)	**< 0.001**
Proteinuria (g/24 h)	2.8 (1.8–4.8)	3.8 (2.0–6.3)	4.8 (2.9–7.3)	7.6 (4.0–11.1)	**< 0.001**
Serum albumin (g/L)	29.6 ± 5.7	29.3 ± 7.0	26.0 ± 5.7	26.3 ± 4.8	**< 0.001**
Microscopic hematuria, n (%)	50 (50.0%)	36 (64.3%)	118 (57.6%)	4 (50.0%)	0.422
Serum creatinine (μmol/L)	61.3 (50.9–72.9)	66.2 (52.8–78.9)	67.3 (55.0–83.0)	133.4 (99.5–174.6)	**< 0.001**
eGFR (ml/min per 1.73m^2^)	123.1 (105.4–151.5)	115.5 (96.9–146.9)	109.8 (85.8–139.5)	49.1 (29.3–70.5)	**< 0.001**
Anti-PLA2R antibody positivity, n (%)	54 (54.0%)	36 (64.3%)	147 (71.4%)	6 (85.7%)	**0.016**
Anti-PLA2R antibody level (U/mL)	105.9 (46.5–195.2)	106.4 (61.6–181.8)	117.8 (51.8–241.1)	299.3 (40.6–818.90)	0.659
Anti-THSD7A antibody positivity, n (%)	2 (2.6%)	0(0.0%)	2 (1.2%)	0 (0.0%)	0.657
Hypertension, n (%)	33 (33.0%)	24 (42.9%)	123 (59.4%)	7 (87.5%)	**< 0.001**
Hemoglobin (g/L)	139.0 (128.0–152.0)	137.0 (130.0–150.0)	136.0 (123.0–147.0)	107.0 (120.8–131.5)	**0.004**

Continuous and normally distributed variables were presented as mean ± SD; continuous and non-normally distributed variables were presented as median, IQR; categorical variables were presented as number (%)

The numbers in boldface are statistically significant

The 32 patients with acute tubulointerstitial injury (Additional file 2: Table S2) were older and presented with higher level of urinary protein excretion, lower level of serum albumin, lower level of eGFR, higher percentage of hypertension, and lower level of hemoglobin.

We found no correlation between anti-PLA2R antibody level and pathological features (*P* > 0.05).

### Treatment responses

Among the 371 patients, 254 (68.5%) patients were treated by immunosuppressive drugs and the others by ACEI/ABRs only. 325 (87.6%) patients achieved remission, including 168 (45.3%) patients of complete remission and 157 (42.3%) patients of partial remission. Nephrotic relapse occurred in 90/325 (27.7%) patients (Table 1).

Univariate Logistic regression analysis showed that the higher level of proteinuria (OR = 1.08, 95%CI 1.02–1.15, *P* = 0.016), the positivity of serum anti-PLA2R antibodies (OR = 4.77, 95%CI 1.83–12.41, *P* = 0.001), and the higher level of serum anti-PLA2R antibodies (OR = 1.05, 95%CI 1.02–1.09, *P* = 0.002) were risk factors to the no-remission response after treatments. Multivariate Logistic regression analysis showed that the positivity of serum anti-PLA2R antibodies (OR = 3.20, 95%CI 1.18–8.72, *P* = 0.023) was an independent risk factors to no-remission (Additional file 3: Table S3). Clinical risk factors were evaluated by Toronto risk score. After adjusting by Toronto risk score, the positivity of anti-PLA2R antibodies (OR = 4.63, 95%CI 1.56–13.70, *P* = 0.006) was still an independent risk factor to no-remission.

All the pathological features did not exert any influence on the treatment response of pMN (*P* > 0.05).

### Kidney outcomes

During a median follow up of 26.9 (13.9–45.4) months, 8/371 (2.2%) patients progressed to ESKD, 67 (18.1%) patients experienced kidney dysfunction with an over 50% decline of eGFR from the baseline at kidney biopsy.

**Fig. 2:**
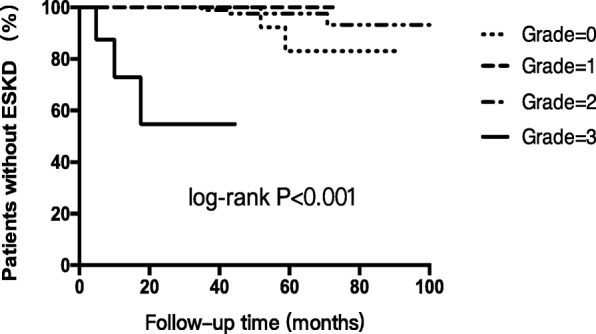
Kaplan-Meier curves analysis for the end stage kidney disease (ESKD) in patients with pMN, with a comparison among the patients with different severity of chronic tubulointerstitial injury. The patients with severe tubulointerstitial injury (grade 3) had worse kidney outcome during follow-up

Kaplan-Meier curve showed that the kidney outcome of ESKD (*P* < 0.001) (Fig. 2) and kidney dysfunction (*P* = 0.079) became worse according to the deterioration of tubulointerstitial injury.

Univariate Cox regression analysis showed that the severity of chronic tubulointerstitial injury (HR = 61.02, 95%CI 7.75–480.57, *P* < 0.001), acute tubulointerstitial injury (HR = 6.94, 95%CI 1.65–29.22, *P* = 0.008), and no-remission after treatments (HR = 14.34, 95%CI 2.98–68.97, *P* = 0.001) were risk factors of ESKD. The higher level of eGFR at baseline on biopsy (HR = 0.97, 95%CI 0.95–0.99, *P* = 0.006) was protective factor of ESKD. Multivariate analysis identified that the severity of chronic tubulointerstitial injury (HR = 25.77, 95%CI 1.27–523.91, *P* = 0.035) and no-remission (HR = 10.17, 95%CI 1.75–59.02, *P* = 0.010) were two independent risk factors of the kidney outcome ESKD (Table 4).

**Table 4:** The risk factors of ESKD in patients with pMN

	Univariate analysis		Multivariate analysis	
	HR (95% CI)	*P*	HR (95% CI)	*P*
Age (increased by 1 year)	1.002 (0.946–1.062)	0.935		
Gender (male)	0.513 (0.230–1.142)	0.102		
Proteinuria (increased by 1 g/24 h)	1.066 (0.924–1.229)	0.380		
Serum albumin (increased by 1 g/L)	0.952 (0.844–1.074)	0.426		
eGFR (increased by 1 ml/min per 1.73m^2^)	0.971 (0.951–0.992)	**0.006**	0.988 (0.957–1.020)	0.464
Anti-PLA2R antibody positivity	36.361 (0.043–30,631.182)	0.296		
Anti-PLA2R antibody level (increased by 20 U/mL)	0.911 (0.746–1.112)	0.360		
Anti-THSD7A antibody positivity	0.049 (0.000-)	0.904		
C3 staining				
negative	ref	–		
1+	1.077 (0.404–2.871)	0.882		
2+	0.991 (0.387–2.535)	0.984		
3+	0.689 (0.209–2.275)	0.542		
MN-stage				
I	ref	–		
II	3.020 (0.585–15.599)	0.187		
III	5.741 (0.514–64.088)	0.156		
Global sclerosis (increased by 1%)	0.577 (0.108–3.082)	0.577		
Crescent (increased by 1%)	0.530 (0.057–4.940)	0.578		
Focal segmental glomerular sclerosis (increased by 1%)	0.644 (0.048–8.623)	0.740		
Chronic tubulointerstitial injury				
Grade = 0	ref	–	ref	–
Grade = 1	0.000 (0.000–0.000)	0.985	0.000 (0.000–0.000)	0.984
Grade = 2	0.445 (0.073–2.702)	0.379	0.460 (0.071–2.971)	0.415
Grade = 3	61.016 (7.747–480.574)	**< 0.001**	25.767 (1.267–523.911)	**0.035**
Acute tubulointerstitial injury	6.941 (1.649–29.217)	**0.008**	0.897 (0.078–9.685)	0.908
Treatments				
ACEI/ARBs	ref	–		
Cyclophosphamide with corticosteroids	3.002 (0.501–18.003)	0.229		
Calcineurin inhibitor w/o corticosteroids	3.479 (0.490–24.722)	0.213		
No remission	14.339 (2.981–68.970)	**0.001**	10.161 (1.749–59.018)	**0.010**

*HR* hazard ratio, *CI* confidence interval

The numbers in boldface are statistically significant

When the clinical risk factors were evaluated by Toronto risk score and enrolled into multivariate Cox regression model together with the severity of chronic tubulointerstitial injury, none of them showed independent prognostic value to ESKD (*P* > 0.05). We found that according to the deterioration of tubulointerstitial injury from grade 0 to 3, the patients presented with higher risk score [0.01 (0.00–0.03) vs. 0.01 (0.00–0.08) vs. 0.02 (0.00–0.30) vs. 0.67 (0.29–0.95), *P* < 0.001]. The correlation (*r* = 0.262, *P* < 0.001) between Toronto risk score and tubulointerstitial injury made the model unstable.

For the kidney dysfunction outcome, univariate Cox regression analysis found that the severity of tubulointerstitial injury (HR = 4.43, 95%CI 1.26–15.59, *P* = 0.021), the older age (HR = 1.02, 95%CI 1.00–1.04, *P* = 0.027), the positivity of serum anti-PLA2R antibodies (HR = 3.19, 95%CI 1.62–6.25, *P* = 0.001), the level of serum anti-PLA2R antibodies (HR = 1.04, 95%CI 1.02–1.06, *P* < 0.001), and no-remission after treatments (HR = 3.86, 95%CI 1.89–7.88, *P* < 0.001) were risk factors of the eGFR reduction over 50%. Toronto risk score was not a risk factor to the eGFR reduction over 50% (HR = 1.01, 95%CI 0.21–4.96, *P* = 0.987). Multivariate regression analysis identified that the positivity of anti-PLA2R antibodies (HR = 2.54, 95%CI 1.26–5.14, *P* = 0.009) and no remission (HR = 3.16, 95%CI 1.46–6.86, *P* = 0.004) were independent risk factors to kidney dysfunction.

## Discussion

In this large consecutive cohort of patients with biopsy-proven pMN, we performed a comprehensive investigation on the pathological parameters of pMN to explore their clinical applications. The intensity of C3 deposit, the stage of membranous lesion, and the severity of tubulointerstitial injury were all associated with the severity of proteinuria and the level of eGFR at the time of kidney biopsy. Only the severity of chronic tubulointerstitial injury was an independent risk factor to the kidney outcome of ESKD and was risk to kidney dysfunction defined as eGFR reduction over 50%. None of the pathological parameters was associated with treatment responses.

We found that the intensity of C3 deposit, the stage of membranous lesion, and the severity of tubulointerstitial injury were all related to the positivity of anti-PLA2R antibodies. This finding implies that the autoantibody was not only a biomarker for clinical presentation of pMN but also a good indicator for the pathological features. There was a correlation between the granular staining of C3 and the proteinuria and kidney function of the patient, which was not observed of IgG deposit. It implies that the deposited anti-PLA2R-IgG may be the beginning of autoimmune disorder and the subsequent complement activation might be more effective to induce kidney pathological injury.

There are many studies focus on the prognostic significance of histopathological parameters of pMN, but a large diversity exists [16, 25]. The reason may be that different parameters were assessed in different studies. In the current study, we investigated all the pathological parameters including immunofluorescence intensity, membranous stages, acute and chronic tubulointerstitial injury, crescent formation, global and focal segmental sclerosis, together with the clinical features including anti-PLA2R antibodies and different treatment regiments and responses, in a large cohort of pMN patients. Then we found that the severity of chronic tubulointerstitial injury was an independent risk factor to the kidney outcome of ESKD. It was also a risk factor to kidney dysfunction. These results were consistent with previous studies [16, 26–31]. Although a Parisian study showed that interstitial fibrosis was not significantly associated with cumulative renal insufficiency [32], when FSGS was removed as a variable, they declared that the interstitial fibrosis showed a significant association with renal insufficiency. In the histopathological report of pMN kidney biopsy, we highly recommend to describe the grades of chronic tubulointerstitial injury, instead of membranous lesion alone. It could provide helpful information to the physicians on kidney prognosis.

We found that severe (> 50% of interstitium involved) chronic tubulointerstitial injury was a risk factor to ESKD. It is also suggested by another Chinese study [28]. However, Chen et al. found that the moderate or severe damage (> 25% of interstitium involved) was risk factor to ESKD [29]. The possible reason for this discrepancy may be that the patients in Chen’s study were restricted to those with nephrotic-range proteinuria (> 3.5 g/day) and CKD stages 2–4. In that ESKD high-risk cohort, the moderate tubulointerstitial injury might show unfavorable outcomes, while in the general pMN patients, more severe tubulointerstitial injury may have risk effect to the outcome of ESKD.

Another risk factor to ESKD was revealed as no-remission in the current study. The clinical composite risk score, Toronto risk score, was also calculated and enrolled in the multivariate Cox regression model. However, since there was close correlation between Toronto risk score and the severity of chronic tubulointerstitial injury, none of them showed independent risk effect to ESKD.

The mechanism remains unclear for that tubulointerstitial damage possesses such an important effect in a glomerular disease. The hypothesis of inflammation-sclerosis is well recognized. The numbers of interstitial macrophages and T cells and the increase of interstitial collagen IV and VI were important factors for pMN prognosis [30]. The tubular epithelial cells can be activated by the cytokines produced by the monocytes/macrophages in the glomerular inflammation lesion and can act as antigen presenting cells to induce the infiltration of T lymphocytes and monocytes/macrophages in the interstitia. The latter may secrete transforming growth factor beta and produce lymphokines to stimulate fibroblasts [33].

We found that none of the pathological parameters showed any effect on treatment responses. Even the patients presented with C3 staining 3+, membranous stage III, acute or severe tubulointerstitial injury, they still had the chance to get complete or partial remission after treatments and to avoid the worse kidney outcomes consequently. This finding may encourage the physicians to give active treatments without scruple of the probably unfavorable effect from the advanced membranous lesion. Different therapies, cyclophosphamides or calcineurin inhibitors, resulted in comparable treatment responses, which was consistent with 2012 KDIGO guideline. The only independent risk factor to no-remission was revealed as the positivity of anti-PLA2R antibodies, together with the possible risk factors as proteinuria and antibody level. These results highlighted the importance of therapeutic strategy to modulate the autoimmune disorders in pMN. Rituximab should be considered for the clearance of autoantibody-producing B cells [10].

There are two advantages in our study. First, all the pathological parameters were comprehensively included into the models to explore their impacts on kidney outcomes in this large cohort, which makes the results much reliable. The second advantage is the enrollment of anti-PLA2R antibody (positivity rate and antibody level) as one of the clinical features, which has been revealed as a prognostic factor to both treatment responses and kidney outcomes [10, 34, 35]. The severity of pathological injury was related to antibody positivity. Multivariate Cox analysis showed that severe chronic tubulointerstitial injury was an independent risk factor to ESKD, but not anti-PLA2R antibody which could be depleted by immunosuppressive therapies. Anti-PLA2R antibody positivity and higher level were risk factors to no-remission. And no-remission was another independent risk factor to ESKD. Thus, anti-PLA2R antibody may display its prognostic role by affecting on treatment response. This finding highlights the necessity of kidney biopsy for pMN, despite the diagnosis based on positive anti-PLA2R antibodies. The information on tubulointerstitial injury and crescent formation could be helpful for evaluating prognosis.

There are limitations in the current study. As a retrospective study, the staining of PLA2R in glomeruli was not available, which might lead to an underestimation of the percentage of PLA2R-related MN. Another limitation was the short time of follow-up. For a slowly progressive glomerular disease like pMN, 7–10 years of follow-up is considered appropriate for the assessment of primary endpoints ESKD [6]. Thus, we added the secondary endpoint as kidney dysfunction defined as the over 50% reduction of eGFR. The unfavorable prognostic effects of tubulointerstitial injury were observed on both endpoints.

## Conclusions

In conclusion, our study identified the chronic damage > 50% of interstitium as an independent predictor of kidney progression to ESKD in pMN. The grade of tubulointerstitial lesions “T” should be important in the histopathological report of pMN.

## Additional files

Additional file 1: **Table S1.** Comparisons of clinical characteristics of pMN patients with different stages of membranous lesion. (DOCX 17 kb)

Additional file 2: **Table S2.** Comparisons of clinical characteristics of pMN patients with and without acute tubulointerstitial injury. (DOCX 20 kb)

Additional file 3: **Table S3.** The risk factors of no-remission in the patients with pMN. (DOCX 21 kb)

## Abbreviations

ACEI: Angiotensin converting enzyme inhibitors
ARB: Angiotensin receptor blocker
C3: Complement 3
CI: Confidence interval
CKD: Chronic kidney disease
eGFR: Estimated glomerular filtration rate
ESKD: End-stage kidney disease
FSGS: Focal glomerular sclerosis
HR: Hazard ratio
IQR: Interquartile range
KDIGO: Kidney Disease: Improving Global Outcomes
OR: Odds ratio
PLA2R: M-type phospholipase A_2_ receptor
pMN: Primary membranous nephropathy
THSD7A: Thrombospondin type 1 domain containing 7A
