# Supplementary material for: Clinical implications of pathological features of primary membranous nephropathy
Source: BMC Nephrol. 2018 Aug 28;19:215. doi: 10.1186/s12882-018-1011-5 (PMC6114049; doi:10.1186/s12882-018-1011-5)
Supplement: Supplementary file 1 — Table S1. Comparisons of clinical characteristics of pMN patients with different stages of membranous lesion. (DOCX 17 kb) [file 12882_2018_1011_MOESM1_ESM.docx]

**Table S1. Comparisons of clinical characteristics of pMN patients with different stages of membranous lesion.**

|  | Stage I (n=193) | Stage II (n=159) | Stage III (n=19) | P |
| --- | --- | --- | --- | --- |
| Age (year) | 55 (46-62) | 51 (43-60) | 50 (33-64) | 0.247 |
| Gender (M/F) | 97/96 | 101/58 | 9/10 | **0.033** |
| Nephrotic syndrome, n (%) | 122 (63.2%) | 119 (74.8%) | 14 (73.7%) | 0.057 |
| Proteinuria (g/24h) | 3.5 (2.1-5.4) | 4.8 (2.8-8.3) | 5.7 (3.4-10.9) | **0.001** |
| Serum albumin (g/L) | 27.9 ± 6.0 | 27.2 ± 6.1 | 25.9 ± 7.8 | 0.279 |
| Microscopic hematuria, n (%) | 99 (51.8%) | 97 (61.8%) | 12 (63.2%) | 0.148 |
| Serum creatinine (μmol/L) | 62.4 (52.2-75.3) | 69.1 (56.0-87.0) | 81.1 (56.7-109.0) | **0.001** |
| eGFR (ml/min per 1.73m^2^) | 116.5 (99.3-146.2) | 109.2 (81.7-140.8) | 97.7 (63.7-146.5) | **0.026** |
| Anti-PLA2R antibody positivity, n (%) | 116 (60.4%) | 112 (70.4%) | 15 (83.3%) | **0.040** |
| Anti-PLA2R antibody level (U/mL) | 105.9 (55.0-207.4) | 123.5 (50.6-250.5) | 77.9 (51.6-173.4) | 0.742 |
| Anti-THSD7A antibody positivity, n (%) | 1 (0.7%) | 3 (2.3%) | 0 (0.0%) | 0.470 |
| Hypertension, n (%) | 90 (46.6%) | 83 (52.2%) | 14 (73.7%) | 0.066 |
| Hemoglobin (g/L) | 138.0 (129.0-148.8) | 135.5 (123.8-149.0) | 127.0 (110.3-140.8) | 0.059 |

Continuous and normally distributed variables were presented as mean ± SD; continuous and non-normally distributed variables were presented as median, IQR; categorical variables were presented as number (%).
