# Supplementary material for: Clinical implications of pathological features of primary membranous nephropathy
Source: BMC Nephrol. 2018 Aug 28;19:215. doi: 10.1186/s12882-018-1011-5 (PMC6114049; doi:10.1186/s12882-018-1011-5)
Supplement: Supplementary file 2 — Table S2. Comparisons of clinical characteristics of pMN patients with and without acute tubulointerstitial injury. (DOCX 20 kb) [file 12882_2018_1011_MOESM2_ESM.docx]

**Table S2.** **Comparisons of clinical characteristics of pMN patients with and without** **acute** **tubulointerstitial injury.**

|  | Without acute tubulointerstitial injury (n=339) | With acute tubulointerstitial injury (n=32) | P |
| --- | --- | --- | --- |
| Age (year) | 52 (43-61) | 60 (55-67) | **0.002** |
| Gender (M/F) | 189/150 | 18/14 | 0.957 |
| Nephrotic syndrome, n (%) | 227 (67.0%) | 28 (87.5%) | **0.017** |
| Proteinuria (g/24h) | 3.8 (2.3-6.1) | 8.3 (4.7-11.7) | **<0.001** |
| Serum albumin (g/L) | 27.9 ± 6.1 | 23.5 ± 5.1 | **<0.001** |
| Hematuria, n (%) | 185 (55.2%) | 23 (71.9%) | 0.069 |
| Serum creatinine (μmol/L) | 63.2 (53.0-77.1) | 99.1 (78.0-152.5) | **<0.001** |
| eGFR (ml/min per 1.73m^2^) | 117.4 (97.8-146.5) | 70.5 (39.2-100.8) | **<0.001** |
| Anti-PLA2R antibody positivity, n (%) | 218 (64.5%) | 25 (80.6%) | 0.070 |
| Anti-PLA2R antibody level (U/mL) | 107.5 (51.2-225.6) | 143.2 (55.8-282.6) | 0.303 |
| Anti-THSD7A antibody positivity, n(%) | 1 (1.1%) | 3 (1.5%) | 0.810 |
| Hypertension, n (%) | 161 (47.5%) | 26 (81.3%) | **<0.001** |
| Hemoglobin (g/L) | 137.0 (126.0-149.0) | 126.0 (105.0-140.0) | **0.019** |

Continuous and normally distributed variables were presented as mean ± SD; continuous and non-normally distributed variables were presented as median, IQR; categorical variables were presented as number (%).
