# Supplementary material for: Clinical implications of pathological features of primary membranous nephropathy
Source: BMC Nephrol. 2018 Aug 28;19:215. doi: 10.1186/s12882-018-1011-5 (PMC6114049; doi:10.1186/s12882-018-1011-5)
Supplement: Supplementary file 3 — Table S3. The risk factors of no-remission in the patients with pMN. (DOCX 21 kb) [file 12882_2018_1011_MOESM3_ESM.docx]

**Table S3.** **The risk factors of no-remission in the patients with pMN.**

|  | Univariate analysis | |  | Multivariate analysis | |
| --- | --- | --- | --- | --- | --- |
|  | OR (95% CI) | P |  | OR (95% CI) | P |
| Age (increased by 1 year) | 0.996 (0.974-1.019) | 0.754 |  |  |  |
| Gender (male) | 1.145 (0.612-2.142) | 0.672 |  |  |  |
| Proteinuria (increased by 1g/24h) | 1.082 (1.015-1.154) | **0.016** |  | 1.023 (0.933-1.122) | 0.627 |
| Serum albumin (increased by 1g/L) | 0.951 (0.901-1.003) | 0.064 |  | 0.970 (0.899-1.047) | 0.970 |
| eGFR (increased by 1ml/min per 1.73m^2^) | 0.993 (0.984-1.001) | 0.097 |  | 0.997 (0.985-1.009) | 0.619 |
| Anti-PLA2R antibody positivity | 4.768 (1.832-12.411) | **0.001** |  | 4.626 (1.562-13.7) | **0.006** |
| Anti-PLA2R antibody level (increased by 20U/mL) | 1.052 (1.019-1.087) | **0.002** |  |  |  |
| Anti-THSD7A antibody positivity | 3.131(0.315-31.118) | 0.330 |  |  |  |
| C3 staining |  |  |  |  |  |
| negative | ref | --- |  |  |  |
| 1+ | 0.589 (0.113-3.062) | 0.529 |  |  |  |
| 2+ | 1.398 (0.306-6.392) | 0.666 |  |  |  |
| 3+ | 2.769 (0.560-13.685) | 0.211 |  |  |  |
| MN-stage |  |  |  |  |  |
| I | ref | --- |  |  |  |
| II | 1.118 (0.586-2.133) | 0.734 |  |  |  |
| III | 2.073 (0.631-6.806) | 0.230 |  |  |  |
| Global sclerosis (increased by 1%) | 1.098 (0.979-1.232) | 0.111 |  |  |  |
| Crescent (increased by 1%) | 1.099 (0.935-1.293) | 0.252 |  |  |  |
| Focal segmental glomerular sclerosis (increased by 1%) | 1.017 (0.840-1.231) | 0.863 |  |  |  |
| Chronic tubulointerstitial injury |  |  |  |  |  |
| Grade=0 | ref | --- |  |  |  |
| Grade=1 | 2.214 (0.758-6.472) | 0.146 |  |  |  |
| Grade=2 | 2.165 (0.914-5.129) | 0.079 |  |  |  |
| Grade=3 | 4.429 (0.750-26.133) | 0.100 |  |  |  |
| Acute tubulointerstitial injury | 2.154(0.874-5.309) | 0.096 |  |  |  |
| Treatments |  |  |  |  |  |
| ACEI/ARBs | ref | --- |  |  |  |
| Cyclophosphamide with corticosteroids | 1.776 (0.846-3.732) | 0.129 |  |  |  |
| Calcineurin inhibitor w/o corticosteroids | 1.271 (0.506-3.193) | 0.609 |  |  |  |

ACEI: angiotensin converting enzyme inhibitors; ARB: angiotensin receptor blocker; OR: Odd ratio; CI: confidence interval.
